# MRI-based measurements of spondylolisthesis and kyphosis in degenerative cervical myelopathy

**DOI:** 10.1186/s12880-023-01151-x

**Published:** 2023-11-09

**Authors:** Eddie de Dios, Mats Laesser, Isabella M. Björkman-Burtscher, Lars Lindhagen, Anna MacDowall

**Affiliations:** 1 Department of Radiology, Institute of Clinical Sciences, Sahlgrenska Academy, University of Gothenburg, Gothenburg, Sweden; 2 Department of Radiology, Sahlgrenska University Hospital, Region Västra Götaland, Bruna stråket 11, Gothenburg, 41345 Sweden; 3 Uppsala Clinical Research Center, Uppsala University, Uppsala, Sweden; 4 Department of Surgical Sciences, Uppsala University, Uppsala, Sweden

**Keywords:** Cervical spine, Magnetic resonance imaging, Spondylolisthesis, Kyphosis, Cervical alignment, Degenerative cervical myelopathy

## Abstract

**Background:**

To provide normative data and to determine accuracy and reliability of preoperative measurements of spondylolisthesis and kyphosis on supine static magnetic resonance imaging (MRI) of patients with degenerative cervical myelopathy.

**Methods:**

T2-weighted midsagittal images of the cervical spine were in 100 cases reviewed twice by one junior observer, with an interval of 3 months, and once by a senior observer. The spondylolisthesis slip (SSlip, mm) and the modified K-line interval (mK-line INT, mm) were assessed for accuracy with the standard error of measurement (SEm) and the minimum detectable change (MDC). Intraobserver and interobserver reliability levels were determined using the intraclass correlation coefficient (ICC).

**Results:**

The SEm was 0.5 mm (95% CI 0.4–0.6) for spondylolisthesis and 0.6 mm (95% CI 0.5–0.7) for kyphosis. The MDC, i.e., the smallest difference between two examinations that can be detected with statistical certainty, was 1.5 mm (95% CI 1.2–1.8) for spondylolisthesis and 1.6 mm (95% CI 1.3–1.8) for kyphosis. The highest reliability levels were seen between the second observation of the junior examiner and the senior observer (ICC = 0.80 [95% CI 0.70–0.87] and ICC = 0.96 [95% CI 0.94–0.98] for SSlip and mK-line INT, respectively).

**Conclusions:**

This study provides normative values of alignment measurements of spondylolisthesis and kyphosis in DCM patients. It further shows the importance of taking measurement errors into account when defining cut-off values for cervical deformity parameters and their potential clinical application in surgical decision-making.

## Background

Degenerative cervical myelopathy (DCM) is caused by spinal canal narrowing due to disc degeneration, osteophyte formation, and enlargement of the ligamentum flavum [1]. The condition may entail cervical malalignment with spondylolisthesis and kyphosis impacting surgical strategy and outcome [2, 3]. Deformity and pathological mobility may contribute to compression or injury of the spinal cord and aggravate myelopathy symptoms [4, 5]. Several surgical treatment options are established for DCM [6], but consensus is limited regarding choice of treatment in different radiological settings [7].

Traditionally, spondylolisthesis and kyphosis have been assessed on lateral x-rays including flexion and extension views in upright position. In clinical practice, however, these deformities are along with myelopathy also assessed on magnetic resonance imaging (MRI) when x-rays are not available or not routinely performed. It is often argued that conventional MRI in a supine neutral position is insufficient to characterize sagittal malalignment compared with lateral x-rays [8–10]. However, as MRI is the most widely used assessment tool due to its superiority in evaluating soft tissues and the spinal cord, it is important to validate MRI-based alignment measurements and to understand MRI-specific limitations prior to defining imaging-based indications for surgery.

MRI-based predictors such as spinal cord compression and intramedullary signal intensity abnormalities have shown association with worse postoperative clinical outcome [11–13]. However, research efforts into the role of sagittal alignment factors have yielded less conclusive results, often in the context of specific surgical methods [14–18]. Comparisons of these studies are further complicated by different thresholds to define spondylolisthesis and kyphosis [10, 19, 20]. Apart from a few studies that have demonstrated good interobserver reliability when using categorical criteria for spondylolisthesis (normal/anterolisthesis/retrolisthesis) and kyphosis (normal/kyphosis) [21, 22], there is little guidance from previous research.

The aims of this study were to provide normative data and to determine the accuracy and reliability of preoperative measurements of spondylolisthesis and kyphosis on supine static MRI of patients with DCM.

## Methods

The study was approved by the Swedish Ethical Review Authority (2017/450, amendment 2019 − 00913). Written informed consent was waived by the authority due to the register-based study design.

### Study design

This is a post-hoc analysis of a previously published retrospective cohort study with 717 patients who underwent surgery for DCM with laminectomy alone or laminectomy with fusion at the 18 major spine units in Sweden from January 2006 to March 2019 [23]. The previous study compared the surgical outcomes between the groups. All patients were included through the Swedish Spine Register (Swespine), which is governed by the Swedish Society of Spinal Surgeons (www.4s.nu) and reports approximately 80% of all spine surgeries in Sweden. Patients were eligible if they were 18 years or older, diagnosed with cervical spinal stenosis and exhibited at least one clinical sign of myelopathy, and treated with laminectomy alone or laminectomy with fusion. Exclusion criteria were previous cervical spine surgery or comorbidities including traumatic spinal injury, spinal infection, inflammatory spondyloarthropathies, neoplastic disease, cardiac disease, neurological disease, or unspecified conditions causing significant pain or gait disturbance. In addition to register-based data, preoperative MRIs were obtained from each hospital for review. In Sweden, preoperative lateral x-rays are not routinely performed in DCM cases and were therefore not retrieved and evaluated in this cohort.

### Radiological evaluation

**Fig. 1:**
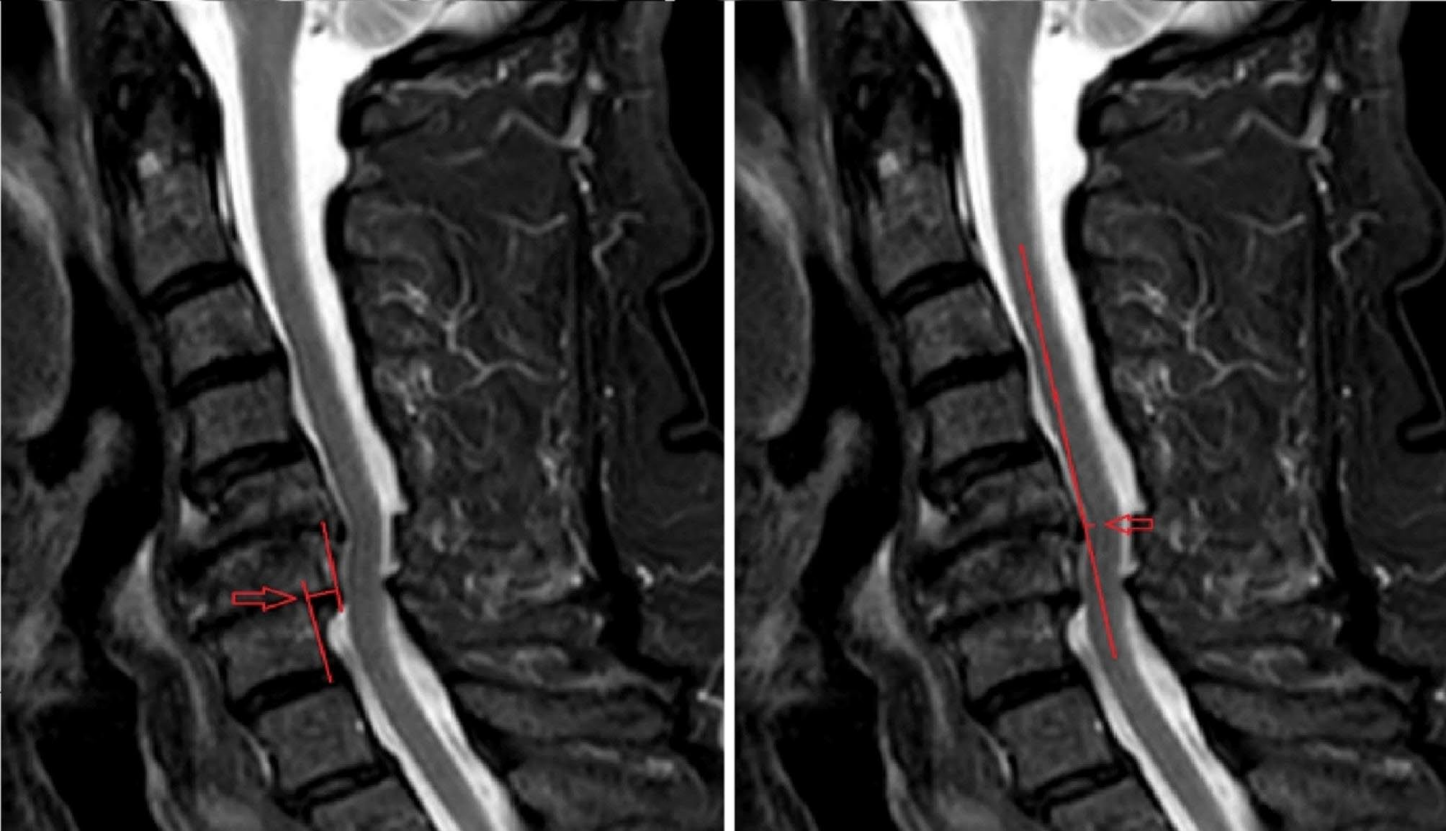
Spondylolisthesis slip (**left**) measured at the disc level between lines drawn along the posterior surface of the two adjacent vertebral bodies with the largest slip and kyphosis (**right**) measured using the modified K-line interval: the minimum interval between a line connecting the midpoints of the spinal cord at the level of the inferior endplates of C2 and C7 and the tip of the anterior compression factor

Preoperative MRI examinations could be retrieved for 487 (68%) of the 717 initially included patients. Among these, 100 MRI examinations were randomly selected for evaluation of spondylolisthesis and kyphosis on T2-weighted midsagittal images of the cervical spine (C2–Th1). Acquisition resolution varied due to different MRI protocols around the country and an observation period of 13 years. The standard slice thickness for 2D sequences was 3–4 mm along with a submillimeter in-plane acquisition resolution and a general reconstruction resolution < 0.5 mm. Spondylolisthesis was measured at the disc level between two lines drawn along the posterior surface of the two adjacent vertebral bodies with the largest slip, i.e., spondylolisthesis slip (SSlip) (Fig. 1) [10]. Kyphosis was measured using the modified K-line interval (mK-line INT): the minimum interval between a line connecting the midpoints of the spinal cord at the level of the inferior endplates of C2 and C7 and the tip of the anterior compression factor (Fig. 1) [24]. Measurements of SSlip and mK-line INT were performed with a precision of 0.1 mm after enhancing the image in the DICOM (Digital Imaging and Communications in Medicine) viewer (Carestream Multimedia Archive). These parameters were independently measured once by an experienced cervical spine surgery consultant as senior observer and twice by a resident with 2.5 years of neurosurgical experience as junior observer (measurement interval of 3 months). The junior observer received instructions from the senior observer before the first reading, but no systematic feedback was later given between the two readings. The observers were blinded to any patient information and to each other.

### Statistical analysis

Each observer’s measurements of spondylolisthesis and kyphosis were summarized with a mean, a standard deviation (SD), and a range for the entire set of 100 single measurements separately for each observer and for the two measurement timepoints for the junior observer. Additionally, the distributions of the senior observer’s measurements of spondylolisthesis and kyphosis were illustrated with bar plots. The mean difference for each intra- and interobserver comparison was tested for significance with the paired *t*-test and a 95% confidence interval (CI) to screen for systematic errors, i.e., whether one observer systematically measured larger or smaller values than the other. Statistical significance was set to *p*-value 0.05 or less. The SD was used as a measure of agreement for mean differences without significant systematic errors. The mean absolute difference (MAD), i.e., the mean of the absolute value of the difference, was also used as a measure of agreement as it incorporates systematic errors. Agreement was also illustrated with Bland-Altman plots.

Accuracy in the study population was determined using the standard error of measurement (SEm) and the minimum detectable change (MDC). SEm, i.e., the typical measurement error, was estimated using a random-effects linear regression model with the two observers’ values (junior observer second measurement and senior observer measurement) as outcome and the patient as the random effect. MDC, the smallest change in a true value that can be detected with 95% statistical confidence, taking the typical measurement error into account, was defined as MDC *= 1.96 x √2 x* SEm. Thus, SEm estimates by how many mm one single measurement can be wrong, whereas MDC represents the smallest difference in mm that can be detected with statistical certainty when a measurement is performed on two separate scoring events on the same radiological examinations. The MDC value can thus be used as a surrogate for the minimum difference between two different examinations, e.g., a baseline examination and a follow-up examination, but will indeed not account for the variability due to differences in technical aspects between two examinations.

The intra- and interobserver reliabilities were estimated with the intraclass correlation coefficient (ICC) and are denoted as IaCC and IeCC, respectively.

The 95% CIs for SEm, MDC, and IaCC/IeCC were computed with 10,000 bootstrap replicates and the percentile method. All statistical analyses were performed in R version 4.0.3 (R Foundation for Statistical Computing, Vienna, Austria).

## Results

**Fig. 2:**
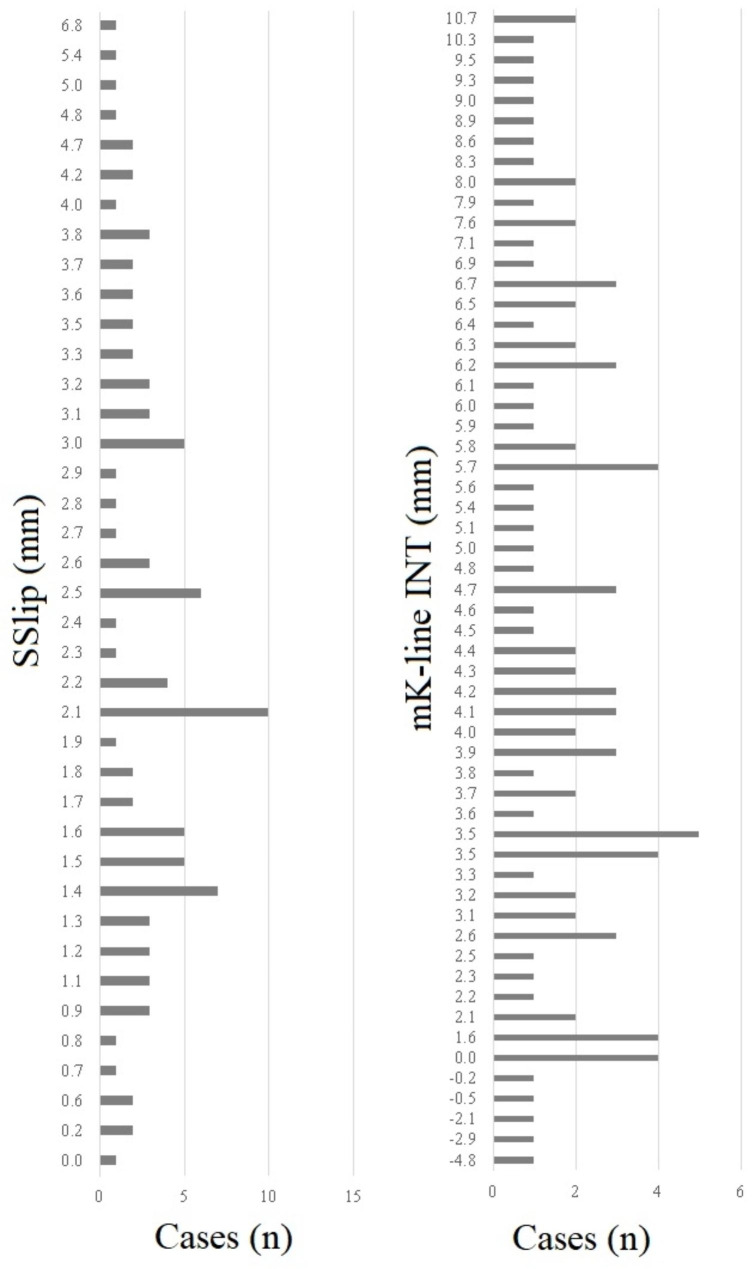
Bar plots illustrating the distribution of the senior observer’s results for spondylolisthesis slip (SSlip) **(left**) and the modified K-line interval (mK-line INT) (**right**)

A descriptive data summary is given in Table 1. Based on the senior measurements, the mean SSlip was 2.3 mm, and the mean mK-line INT was 4.5 mm. The bar plots show the distribution of spondylolisthesis and kyphosis measurements by the senior observer (Fig. 2).

**Table 1:** Descriptive data summary of the spondylolisthesis slip (SSlip) and the modified K-line interval (mK-line INT) measurements from each observer in the evaluated 100 cases

Variable	Junior, baseline measurement	Junior, measurement after 3 months	Senior
	mean ± standard deviation (range)		
**SSlip (mm)**	2.0 ± 1.6 (0.0–7.4)	2.5 ± 1.2 (0.0–5.6)	2.3 ± 1.2 (0.0–6.8)
**mK-line INT (mm)**	4.7 ± 3.2 (-5.4–13.9)	4.4 ± 2.9 (-5.0–11.2)	4.5 ± 2.8 (-4.8–11.7)

**Fig. 3:**
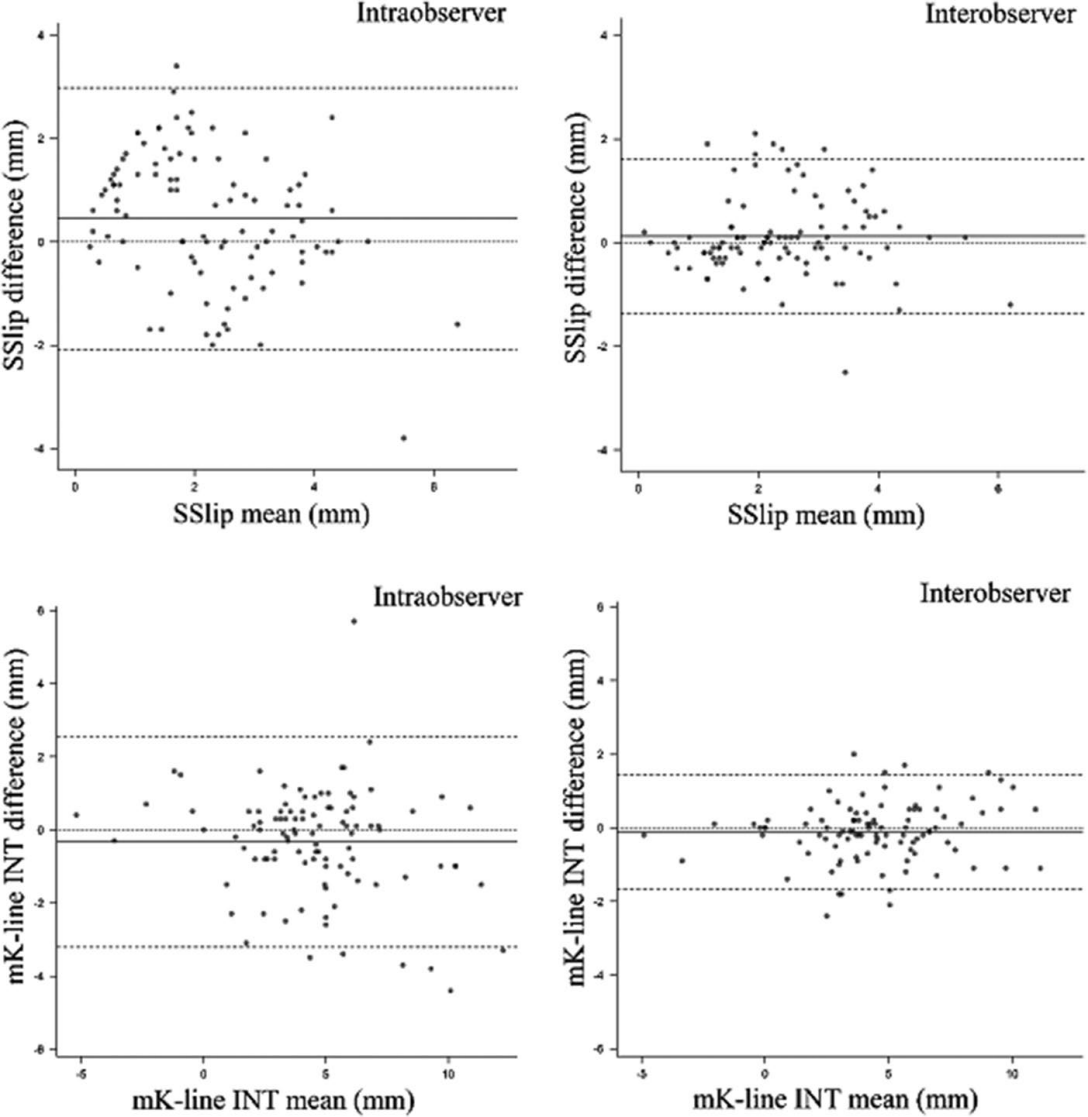
Bland-Altman plots for spondylolisthesis slip (SSlip) (**upper row**) and for modified K-line interval (mK-line INT) (**lower row**) for intraobserver agreement (**left**) and interobserver agreement between the second observation of the junior examiner and the senior observer (**right**). Dotted line: perfect agreement; solid line: mean difference; dashed lines: limits encompassing 95% of the points. Note: scale differences of x-axis between intra- and interobserver agreement

The smallest systematic measurement errors were seen between the second measurements of the junior examiner and the senior measurements (mean difference 0.1 mm [95% CI -0.03–0.3] for SSlip and mean difference − 0.1 mm [95% CI -0.3–0.04] for mK-line INT). Further details are shown in Table 2. The best overall agreement levels were also seen between the second junior and the senior measurements, as also underlined by the more aggregated distribution of observation points in the Bland-Altman plots for interobserver compared with intraobserver SSlip and mK-line INT (Fig. 3).

Based on the measurements performed by the junior observer (second reading) and the senior observer together, the SEm was 0.5 mm (95% CI 0.4–0.6) for SSlip and 0.6 mm (95% CI 0.5–0.7) for mK-line INT. Based on these measurements, the MDC, i.e., the smallest detectable change between two measurements, was 1.5 mm (95% CI 1.2–1.8) for SSlip and 1.6 mm (95% CI 1.3–1.8) for mK-line INT (Table 2).

The highest reliability levels were seen between the second observation of the junior examiner and the senior observer (IeCC = 0.80 [95% CI 0.70–0.87] for SSlip and IeCC = 0.96 [95% CI 0.94–0.98] for mK-line INT) (Table 2).

**Table 2:** Results from the inter- and intra-agreement analysis for the two observers presented separately for spondylolisthesis and kyphosis

Comparison	Mean difference (95% CI)	*p*-value	SD	MAD	SEm (95% CI)	MDC (95% CI)	ICC (95% CI)
***Spondylolisthesis slip (SSlip; mm)***							
**Junior T**_**0**_ vs. **Senior**	-0.3 (-0.6 – -0.04)	**0.024**	1.4	1.2	1.0 (0.9 − 1.2)	2.8 (2.5–3.2)	0.49 (0.30–0.63)
**Junior T**_**3m**_ vs. **Senior**	0.1 (-0.03–0.3)	0.11	0.8	0.5	0.5 (0.4–0.6)	1.5 (1.2–1.8)	0.80 (0.70–0.87)
**Junior T**_**3m**_ vs. **Junior T**_**0**_	0.5 (0.2–0.7)	**< 0.001**	1.3	1.1	1.0 (0.8–1.1)	2.7 (2.3–3.0)	0.56 (0.41–0.67)
***Kyphosis measured with modified K-line interval (mK-line INT; mm)***							
**Junior T**_**0**_ vs. **Senior**	0.2 (-0.1–0.5)	0.11	1.3	0.9	0.9 (0.7–1.1)	2.6 (2.1–3.2)	0.90 (0.84–0.94)
**Junior T**_**3m**_ vs. **Senior**	-0.1 (-0.3–0.04)	0.15	0.8	0.6	0.6 (0.5–0.7)	1.6 (1.3–1.8)	0.96 (0.94–0.98)
**Junior T**_**3m**_ vs. **Junior T**_**0**_	-0.3 (-0.6 – -0.03)	**0.030**	1.5	1.1	1.1 (0.9–1.3)	3.0 (2.4–3.5)	0.88 (0.81–0.93)

CI, confidence interval; ICC, intraclass correlation coefficient; MAD, mean absolute difference; MDC, minimum detectable change; SD, standard deviation; SEm, standard error of measurement; T_0_, baseline measurement; T_3m_, measurement after 3 months

## Discussion

This validation study of supine static MRI measurements of spondylolisthesis and kyphosis as defined by SSlip and mK-line INT provides normative data of a national, surgically treated DCM cohort, showing that the average patient did not exhibit strong radiological signs of spondylolisthesis or kyphosis.

The highest agreement levels for spondylolisthesis and kyphosis measurements were seen between the second observation of the junior examiner and the senior observer. After three months, there were no statistically significant differences between the measurements of the junior and senior observers, whereas the intraobserver differences for the junior observer were significant for both SSlip and mK-line INT, indicating a substantial learning curve-effect for the junior observer when using the senior observer as ground truth. As the junior observer was blinded to the senior observer, without receiving any systematic feedback from the senior observer between the two readings, the learning curve is most probably explained by the increased experience of performing the measurements during the first reading.

The presented SEm and MDC values for SSlip and mK-line INT indicate that these measurements can be performed with high accuracy. SEm and MDC values were calculated using data from the senior observer and from the second reading of the junior observer, as these were regarded as more reliable, with IeCC being ‘high’ for spondylolisthesis and ‘very high’ for kyphosis, whereas the IaCC values were negatively impacted by the learning curve-effect.

A SEm of 0.5 mm for SSlip and 0.6 for mK-line INT suggests that a definition of these entities should account for a measurement error of at least 0.5 mm. However, when taking the SEm CIs and the acquisition in-plane resolution of < 1 mm into account, it is reasonable to suggest that measurements in clinical practice should not be performed with a precision lower than 1 mm. Similarly, the MDC values suggest that an improvement or deterioration in SSlip or mK-line INT has occurred when there is a change of at least 2 mm between two MRI examinations. It is possible that even smaller SEm and MDC values could be obtained if calculations were to be based on intra-observations of one senior observer. Even so, it is unlikely that these differences would change the estimated measurement error of 1 mm for one examination or the smallest detectable change of 2 mm between two examinations. As the MDC values were calculated based on the same radiological examinations evaluated by two observers, it is also probable that the MDC values would be slightly higher if calculated based on two different examinations. This is also an argument for suggesting a change of at least 2 mm as a cut-off for possible measurement errors between two examinations as opposed to the MDC values of 1.5 and 1.6 mm for SSlip and mK-line INT, respectively, when calculated based on separate scoring events on the *same* examination.

More importantly, these results only put forward minimum cut-off values from a technical point of view, whereas clinical cut-off values should be based on their predictive importance and be useful in surgical decision-making. At the same time, our findings highlight the importance of taking measurement errors into account as a measured change between two examinations in mK-line INT of 4 mm could correspond to a true change ranging from 2 to 6 mm. Therefore, when using a malalignment as an argument for a certain surgical intervention or when analyzing research findings that use thresholds of these magnitudes, these aspects need close attention.

The mK-line INT has previously proven to be a useful MRI tool in predicting postoperative outcome after DCM surgery [24], but for MRI measurements of spondylolisthesis, there is currently no clear evidence to support a predictive value, despite spondylolisthesis often being associated with more severe DCM [25]. In the lumbar spine, conventional MRI has shown a sensitivity of 78% for detecting degenerative spondylolisthesis. Although not as high as the sensitivity of 98% for standing lateral radiographs in the same study, MRI will still be useful to find most rigid slips that surgeons consider as relevant but will indeed miss those slips that have a translational component between supine and standing position. Furthermore, the interobserver agreement was higher in the supine MRI group compared with the x-ray group (kappa statistic (κ) = 0.91 versus κ = 0.80) [26]. In addition, the cost-effectiveness of using plain radiographs has not been demonstrated. Nonetheless, a comparison of the clinical utility between weight-bearing x-rays and conventional MRIs is outside the scope of this article. Such a comparison would also require further elucidation of the actual impact of sagittal malalignment on clinical outcome, which is still a matter of ongoing debate [27, 28].

A problematic area is to what degree of precision alignment parameters should be measured. Considering a reconstructed in-plane resolution of < 0.5 mm and a routinely used sagittal slice thickness of 3 mm, one could argue against a measurement accuracy of 0.1 mm, not least due to potential partial volume effects. We decided to consistently set the degree of measurement precision to 0.1 mm, to allow more nuanced results in the analysis compared to if we had used 1 mm as measurement accuracy. However, we strongly advise not to recommend clinical thresholds for the measures investigated with a measurement accuracy below 1 mm.

Mimicking a clinical situation but presenting a limitation, observers were free to choose any of the provided sagittal slices as the midsagittal slice and the choice was not documented. This could for several reasons be an important explanation for some of the observed discrepancies between observers: (1) two or more adjacent sagittal slices could be regarded as or correspond to the midsagittal slice, (2) thinner slices might increase the possibility of observers choosing different midsagittal slices, (3) bias towards choosing the easiest slice to assess, (4) distortions from coronal deformities such as scoliosis, (5) the partial volume effects, and (6) risk of choosing different levels to measure the maximum vertebral slippage when measuring SSlip or the most compressive anterior factor when measuring the mK-line INT. Further, although the observers were asked to use the posterior wall of the vertebral body as the reference line for SSlip measurements, there were no specific instructions given on how to differentiate between spondylophytes and the vertebral body. In clinical practice, slippages or compressive factors might be documented for several levels, whereas in this study observers were asked to identify the most severely affected level, adding another potential source of bias. In most cases, however, there is little doubt as to what level is the most pathological. The focus of this study was to provide normative data and more knowledge on the internal validity of MRI measurements, and we therefore interpret these uncertainties as natural elements that are incorporated in an agreement study. However, when defining malalignment entities such as spondylolisthesis and kyphosis for clinical guidelines, it is important to take these considerations into account.

## Conclusions

This study provides normative values of alignment measurements of spondylolisthesis and kyphosis on preoperative supine static MRI in a cohort of DCM patients. In this clinical cohort, accuracy was high in terms of SEm and MDC. Interobserver reliability was high for SSlip and mK-line INT and a clear learning curve was seen from an intraobserver evaluation of a junior observer. Our study shows the importance of taking measurement errors into account when defining cut-off values for cervical deformity parameters that can impact surgical decision-making.

## Data Availability

Support data can be made available to a limited extent and upon written request in anonymized form, subject to approval by relevant entities such as the data owner and relevant registers. Furthermore, legal rules and agreements must be followed. Image data is excluded.

## Abbreviations

DCM: Degenerative cervical myelopathy
MRI: Magnetic resonance imaging
SSlip: Spondylolisthesis slip
mK-line: INT modified K-line interval
SD: Standard deviation
CI: Confidence interval
MAD: Mean absolute difference
SEm: Standard error of measurement
MDC: Minimum detectable change
ICC: Intraclass correlation coefficient
IaCC: Intraclass intraobserver correlation coefficient
IeCC: Intraclass interobserver correlation coefficient
